# Structure of *Rhodococcus equi* virulence-associated protein B (VapB) reveals an eight-stranded antiparallel β-barrel consisting of two Greek-key motifs

**DOI:** 10.1107/S2053230X14009911

**Published:** 2014-06-18

**Authors:** Christina Geerds, Jens Wohlmann, Albert Haas, Hartmut H. Niemann

**Affiliations:** a Department of Chemistry, Bielefeld University, Universitaetsstrasse 25, 33615 Bielefeld, Germany; b Institute for Cell Biology, University of Bonn, Ulrich-Haberland Strasse 61a, 53121 Bonn, Germany

**Keywords:** antiparallel β-barrel, Greek-key motif, Vap protein family, single-wavelength anomalous dispersion, *Rhodococcus equi*

## Abstract

Members of the virulence-associated protein (Vap) family from the pathogen *Rhodococcus equi* regulate virulence in an unknown manner. They do not share recognizable sequence homology with any protein of known structure. VapB and VapA are normally associated with isolates from pigs and horses, respectively. To contribute to a molecular understanding of Vap function, the crystal structure of a protease-resistant VapB fragment was determined at 1.4 Å resolution. The structure was solved by SAD phasing employing the anomalous signal of one endogenous S atom and two bound Co ions with low occupancy. VapB is an eight-stranded antiparallel β-barrel with a single helix. Structural similarity to avidins suggests a potential binding function. Unlike other eight- or ten-stranded β-barrels found in avidins, bacterial outer membrane proteins, fatty-acid-binding proteins and lysozyme inhibitors, Vaps do not have a next-neighbour arrangement but consist of two Greek-key motifs with strand order 41238567, suggesting an unusual or even unique topology.

## Abstract 2

The structure of VapB, a member of the Vap protein family that is involved in virulence of the bacterial pathogen *R. equi*, was determined by SAD phasing and reveals an eight-stranded antiparallel β-barrel similar to avidin, suggestive of a binding function. Made up of two Greek-key motifs, the topology of VapB is unusual or even unique.

## 1. Introduction   

*Rhodococcus equi* is a Gram-positive facultative intracellular pathogen mainly of young foals, but also of other animal species and of human AIDS patients. *R. equi* is closely related to mycobacteria such as *Mycobacterium tuberculosis* and causes life-threatening pulmonary and extra-pulmonary infections (Vázquez-Boland *et al.*, 2013 ▶; von Bargen & Haas, 2009 ▶).

Epidemiological studies have shown that infection of different animal species is closely coupled to the possession of certain types of virulence-associated plasmids by the infecting bacteria. The plasmid type typically found in equine isolates mediates production of the immunodominant secreted protein VapA (virulence-associated protein A; Sekizaki *et al.*, 1995 ▶). Porcine isolates typically produce the allelic variant VapB (Takai *et al.*, 2000 ▶). *vapA* and *vapB* have never been described together in a single bacterial isolate. VapA production is absolutely required for virulence phenotypes in a mouse infection model and in murine macrophages (Jain *et al.*, 2003 ▶) and it is also a prerequisite for the neutralization of phagosome and lysosome pH in infected macrophages (von Bargen *et al.*, 2009 ▶). An additional ten *vap* genes and three *vap* pseudo-genes identified on the *vapA*- and *vapB*-encoding virulence plasmids seem to have only a minor role (if any) in virulence (Jain *et al.*, 2003 ▶). The amino-acid sequence similarities between all Vap proteins according to *ClustalW* are typically in the 30–50% identity range, with the 78% identity score between VapA and VapB being particularly high (Letek *et al.*, 2008 ▶).

The precise mechanisms of how Vaps support *R. equi* virulence is unknown, particularly as there is apparently no protein to be found in current amino-acid sequence databases that has a meaningful sequence identity to VapA and for which the function has been annotated. We reasoned that establishing the three-dimensional structure of a Vap protein would possibly hint at the putative function(s) of the Vap family. Here, we present the crystal structure at 1.4 Å resolution of the 12 kDa C-terminal protease-resistant core of recombinantly produced VapB.

## 2. Materials and methods   

### 2.1. Protein expression and purification   

Mature *R. equi* VapB (residues 36–197 lacking the signal sequence) was generated by PCR from the sequenced virulence-associated plasmid of strain PAM 1593 (Letek *et al.*, 2008 ▶). The PCR primers were constructed in such a way that they generated an N-terminally His_6_-tagged VapB protein when cloned into the pETite N-His Kan vector (BioCat, Heidelberg, Germany) by ‘in-fusion reaction’, according to the manufacturer’s manual. The primers were VapB_his-6_forward, 5′-CATCATCACCACCATCACGTGCTGGATTCCGGAGGCGGC-3′, and VapB_his-6_reverse, 5′-GTGGCGGCCGCTCTATTATTATGCAACCTCCCAGTTGTG-3′. Protein expression was induced in *Escherichia coli* BL21 (DE3) cells at an OD_600_ of 0.76 with 1 m*M* IPTG for 4 h at 37°C. Protein purification was carried out at 4°C. The cell pellet from 2 l of bacterial culture was resuspended in 40 ml lysis buffer (50 m*M* NaH_2_PO_4_, 300 m*M* NaCl, 10 m*M* imidazole pH 7) plus one EDTA-free cOmplete protease-inhibitor tablet (Roche) and 3 µl DNase I (40 mg ml^−1^) and lysed by three passes through a French pressure cell. The lysate was cleared by centrifugation and the supernatant was applied onto 10 ml Ni–NTA Sepharose equilibrated in lysis buffer. The resin was washed with 3 × 30 ml lysis buffer (pH 7) containing 10 m*M*, 20 m*M* or 40 m*M* imidazole. The target protein was eluted with lysis buffer including 500 m*M* imidazole (pH 7). The eluate was dialyzed against 2 l phosphate-buffered saline (PBS) twice. The yield was roughly 80 mg protein per litre of bacterial culture. For crystallization, the protein was digested with proteinase K (Roth, product No. 7528.1). 40 mg of VapB were digested with 80 µg proteinase K (500:1 protein:protease mass ratio) in 40 ml PBS for 90 min at 37°C. The reaction was stopped with 400 µl PMSF (phenylmethylsulfonyl fluoride) (17 mg ml^−1^ in ethanol; 100 m*M*) on ice for 20 min and then the solution was dialyzed against 1 l buffer *A* (25 m*M* Tris, 20 m*M* NaCl pH 7), plus 1 ml PMSF (100 m*M* in ethanol) overnight with a protein recovery of about 50% (20.6 mg). Proteinase-K-digested VapB was further purified by anion-exchange chromatography over Source Q (GE Healthcare) resin equilibrated in buffer *A*. The protein was eluted by a salt gradient to 1 *M* NaCl. SDS–PAGE analysis revealed that proteinase K digestion had resulted in two fragments of very similar molecular mass, of which the slightly heavier fragment eluted earlier. Early peak fractions were pooled, dialyzed against buffer *A* overnight, concentrated to 16.8 mg ml^−1^ using Vivaspin concentrators (Sartorius) and frozen in small aliquots. The recovery after proteinase K digestion and purification was about 12.5% (5 mg).

### 2.2. Crystallization   

Crystals grew in a sitting-drop vapour-diffusion setup in the initial sparse-matrix screen (condition G4 of The JCSG Core Suite II; 10 m*M* CoCl_2_, 100 m*M* sodium acetate pH 4.6, 1.0 *M* hexanediol) with 10 mg ml^−1^ protein in buffer *A* at 20°C with a drop consisting of 1 µl protein solution and 0.5 µl reservoir solution. Crystals were observed after 23 weeks (the previous inspection being at 15 weeks), presumably well after the crystallization drop had reached equilibrium. Single crystals could easily be isolated for mounting from a bundle of thick (40–50 µm) needles reaching 200–300 µm in length. The crystal used for phasing was cryoprotected with 10 m*M* CoCl_2_, 100 m*M* sodium acetate pH 4.6, 1.1 *M* 1,6-hexanediol, 20% glycerol. The crystal used for high-resolution data collection was cryoprotected with 10 m*M* CoCl_2_, 100 m*M* sodium acetate pH 4.6, 2.3 *M* 1,6-hexanediol. Both crystals were flash-cooled in liquid nitrogen.

### 2.3. Data collection and processing   

Data sets were collected from two different crystals from the same crystallization drop. High-redundancy data for SAD (single-wavelength anomalous dispersion) phasing were collected on a Super Nova diffractometer (Agilent) equipped with a 40 W copper sealed-tube microsource and an Atlas CCD detector. High-resolution data for refinement were collected on ESRF beamline ID29 at a wavelength of 0.91985 Å on a PILATUS 6M detector. All data were indexed and integrated with *XDS* and scaled with *XSCALE* (Kabsch, 2010 ▶). Data-collection and processing statistics are given in Table 1 ▶.

### 2.4. Structure solution and refinement   

#### 2.4.1. Substructure solution   

The substructure was solved with *SHELXD* (Sheldrick, 2010 ▶) run through the graphical user interface *HKL*2*MAP* (Pape & Schneider, 2004 ▶) for several thousand trials with different high-resolution cutoffs, and between two and four requested sites. Equivalence of the resulting substructures was checked with *phenix.emma* (Adams *et al.*, 2010 ▶). Solutions with CC_all_ and CC_weak_ of around 50 and 30, respectively, yielded three consistent sites.

#### 2.4.2. Density modification and polyalanine model   

Density modification was carried out with the β-test version of *SHELXE* including autotracing. *SHELXE* (Sheldrick, 2010 ▶) was run with 20 cycles of density modification, a solvent fraction of 0.45 and a high-resolution cutoff for the native data of 1.9 Å (the synchrotron data only became available later) for 200 cycles of autotracing. The best autotracing cycle resulted in 84 out of 113 residues in six chains. The resulting structure had a significant amount of secondary structure and revealed a β-barrel with a single helix.

#### 2.4.3. Model completion and refinement and structure analysis   

The resulting polyalanine structure and phases were fed into *phenix.autobuild* (Adams *et al.*, 2010 ▶; Terwilliger *et al.*, 2008 ▶) along with the amino-acid sequence for automatic model completion using default settings. The resulting model had 96 out of 113 residues (84%) built in sequence and *R*_work_ of 23.51% and *R*_free_ of 26.02%. This model was completed manually in *Coot* (Emsley *et al.*, 2010 ▶). For the high-resolution synchrotron data, free-*R* flags were copied from the data set used for phasing and extended. The model was refined with *REFMAC*5 (Murshudov *et al.*, 2011 ▶) using TLS refinement with 12 TLS groups that had been determined with the *TLSMD* server (Painter & Merritt, 2006 ▶). Refinement statistics are given in Table 2 ▶. The oligomeric state was determined with *PISA* (Krissinel & Henrick, 2007 ▶). Structural similarities were identified with the *DALI* server (Holm & Rosenström, 2010 ▶) and *PDBeFold* (Krissinel & Henrick, 2004 ▶). *PDBeFold* was run with default settings except setting ‘lowest acceptable match’ to 60% for both query and target. Figures were prepared with *PyMOL* (DeLano, 2002 ▶). Sequences were aligned with *MAFFT* (Katoh & Standley, 2013 ▶). Alignment figures were prepared with *ESPript* (Gouet *et al.*, 2003 ▶).

## 3. Results and discussion   

### 3.1. Structure determination of the highly conserved protease-resistant Vap core   

We first tried to crystallize VapA, as its role in virulence has been most clearly documented. Full-length VapA was degraded into a stable fragment, presumably by trace amounts of proteases remaining after purification. In an attempt to completely degrade purified VapA to serve as negative control in cellular assays, we had fortuitously found that VapA has a proteinase-K-resistant core consisting of the highly conserved C-terminus (Supplementary Fig. S1^1^). We purified this proteinase-K-resistant fragment for crystallization, but did not obtain any diffracting crystals. Therefore, we turned to VapB, the Vap most similar to VapA. Again, full-length VapB was unstable and did not crystallize. We used proteinase K digestion to remove the N-terminus. Edman sequencing (Proteome Factory, Berlin) revealed that the resulting 12 kDa stable fragment started at amino acid Glu85. We will refer to this fragment as VapB throughout this paper. Within the proteinase-K-resistant domains, the VapA and VapB amino-acid sequences are 88% identical plus 6% highly conserved. VapB crystallized in a condition containing 10 m*M* CoCl_2_ from a commercial screen. We collected a high-redundancy data set on a copper sealed-tube diffractometer (Table 1 ▶). The anomalous signal originating from the S atom of the single methionine and two low-occupancy (∼25%) Co^2+^ ions was sufficient to solve the phase problem by SAD. Clear electron density is visible for residues Glu87–Val196 with some density for residues Gln86 and Ala197. Apparently, there was no or incomplete cleavage of the five C-terminal VapB residues although they are accessible and in a loop conformation.

### 3.2. Structure of VapB   

**Figure 1:**
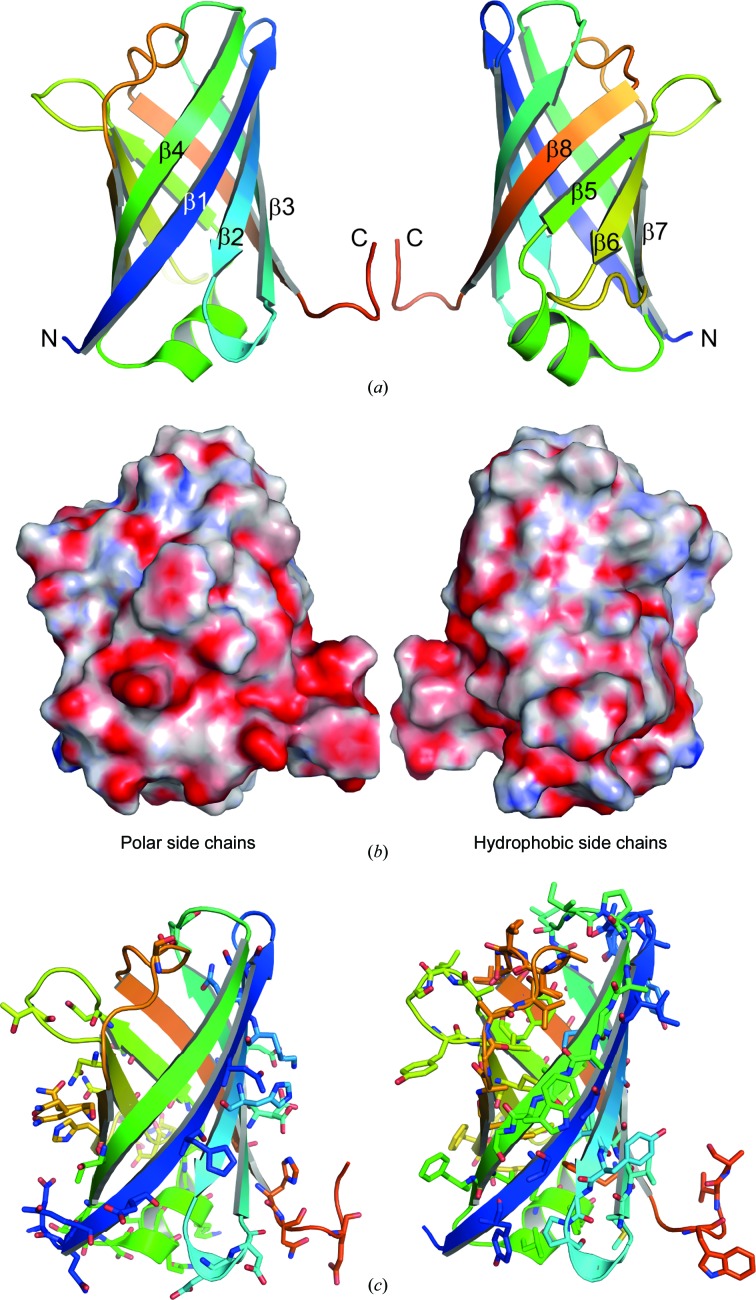
(*a*) Cartoon representation of VapB coloured from dark blue to red from the N-terminus to the C-terminus. The two views are rotated 180° around a vertical axis. (*b*) Electrostatic potential of VapB oriented as in (*a*) and coloured on a scale from −15 (red) to +15 (blue) *kT*/e. (*c*) Distribution of hydrophilic (left) and hydrophobic (right) residues in VapB oriented as in the left of (*a*).

VapB forms a monomeric eight-stranded antiparallel β-barrel with a single helix (Fig. 1 ▶ *a*). We refer to the molecule as oriented in Fig. 1 ▶(*a*) with both termini and the helix at the bottom and the turn connecting strands 1 and 2 and the loops between strands 5 and 6 or 7 and 8 located at the top. The distribution of hydrophobic and polar residues on the VapB surface is not uniform. The bottom is enriched in polar side chains, including eight negatively and two of the three positively charged residues of VapB, while the top is more hydrophobic (Figs. 1 ▶ *b* and 1 ▶ *c*). Conserved surface-exposed residues are presumably relevant for the fold and often form hydrogen bonds *via* their side chains, *e.g.* Tyr102, Tyr143 or Asn159 and Asn161 (Supplementary Fig. S1). Vaps contain many glycine residues that are very well defined in the electron density. They often locate to β-strands and are conserved for steric reasons, assuming backbone conformations that are not accessible to nonglycine residues, or because the addition of only a C^β^ atom would result in clashes (Supplementary Fig. S1). It is questionable whether VapF, another member of the Vap family, can form a stable protein with the same fold, as it lacks highly conserved sequences forming strands 7 and 8 (Supplementary Fig. S2).

A search for similar structures with *DALI* returns mostly other eight-stranded and some ten-stranded β-barrels but no highly significant hits (*Z*-scores < 6; sequence identity < 15%). Similar structures include bacterial outer membrane proteins (Schulz, 2002 ▶), proteins from the avidin/streptavidin superfamily (Livnah *et al.*, 1993 ▶; Weber *et al.*, 1989 ▶) and cytoplasmic fatty-acid-binding proteins (Zimmerman & Veerkamp, 2002 ▶). *PDBeFold* identified bacterial lysozyme inhibitors (Leysen *et al.*, 2011 ▶; Yum *et al.*, 2009 ▶) as top hits.

**Figure 2:**
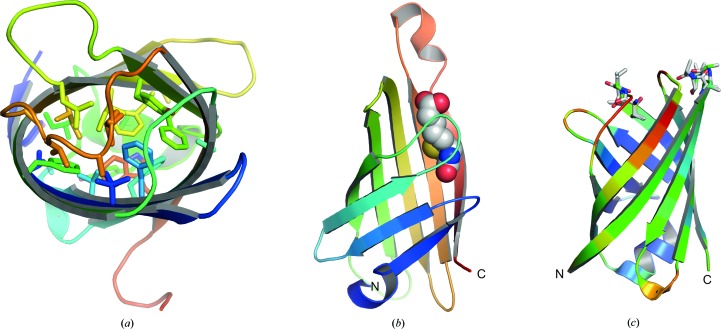
(*a*) View from the top down the axis of the VapB β-barrel. Side chains of residues forming the hydrophobic core are represented as sticks. (*b*) Cartoon representation of streptavidin (PDB entry 1stp; Weber *et al.*, 1989 ▶) coloured from dark blue to red from the N-terminus to the C-terminus. Biotin is shown as spheres. (*c*) VapB coloured by *B* factor (blue, low; red, high) and oriented as streptavidin with respect to strand β1 (note that the structural alignment generated by *DALI* is different). The loops at the top exhibit high *B* factors and double backbone conformations for residues that are represented as sticks.

VapB has a tightly packed core sealed at the bottom by the helix (Fig. 2 ▶ *a*). There is neither a central pore typical of outer membrane porins nor is there a central cavity typical for fatty-acid-binding proteins. Functional similarity might be highest to avidins. Avidins bind biotin in a groove at the top of the molecule, when oriented like VapB (Fig. 2 ▶ *b* ; Livnah *et al.*, 1993 ▶; Weber *et al.*, 1989 ▶). The loops at the top of VapB have higher *B* factors, indicating flexibility (Fig. 2 ▶ *c*). The connections between strands 1 and 2 and strands 7 and 8 (residues 99–101 and 181–184, respectively) display double conformations. It is conceivable that these turns/loops move to open a mainly hydrophobic binding site formed by the top of the VapB hydrophobic core. A function of VapB in binding some kind of ligand thus seems possible.

### 3.3. VapB consists of two Greek-key motifs with unprecedented topology   

**Figure 3:**
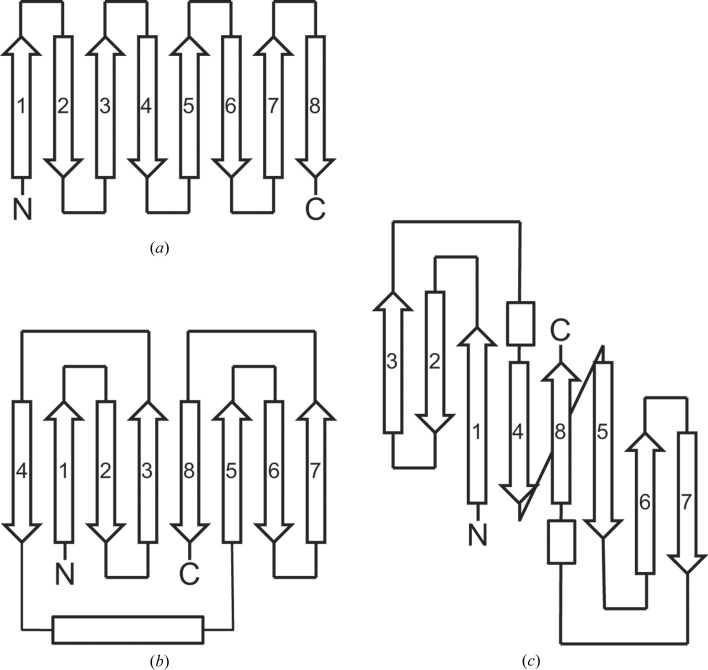
Topology diagrams of eight-stranded antiparallel β-barrels (*a*) with next-neighbour connections as found in outer membrane proteins, avidins and lysozyme inhibitors; (*b*) two Greek-key motifs as present in VapB; (*c*) two Greek-key motifs as described for RPB8 (adapted from Krapp *et al.*, 1998 ▶).

While VapB is structurally related to bacterial outer membrane proteins, avidins, fatty-acid-binding proteins and lysozyme inhibitors, with root-mean-square deviations of around 3 Å for some 70 aligned C^α^ atoms, the topology of VapB is quite unusual. Outer membrane proteins, avidins, fatty-acid-binding proteins and lysozyme inhibitors all are antiparallel β-barrels in which each strand is directly linked to its neighbour (Fig. 3 ▶ *a*). In contrast, the VapB β-barrel consists of two Greek-key motifs (Hutchinson & Thornton, 1993 ▶) with strands arranged in the order 41238567 and a helix connecting strands 4 and 5 (Fig. 3 ▶ *b*). Greek-key motifs are common in five- or six-stranded β-barrels and in β-sandwich structures (Zhang & Kim, 2000 ▶), for example the β- and γ-crystallins with two Greek-key motifs that form two four-stranded sheets (Jaenicke & Slingsby, 2001 ▶). A comprehensive review of Greek-key motifs in β-barrels stated that there is only one eight-stranded β-barrel known that contains Greek keys (Zhang & Kim, 2000 ▶). This protein (RNA polymerase subunit RPB8; PDB entry 1a1d; Krapp *et al.*, 1998 ▶) has strand order 32148567 (Fig. 3 ▶ *c*) and is structurally unrelated to VapB. While we cannot exclude the fact that an eight-stranded β-barrel with the strand order of VapB has been described before, such a topology is not found among the top hits of *DALI* or *PDBeFold*.

In conclusion, we have determined the structure of VapB as representative of the *R. equi* Vap family. The Vaps fold into two Greek-key motifs forming an eight-stranded β-barrel with an unusual, if not unprecedented, topology. The arrangement of strands is different from β-barrels with next-neighbour connections present in outer membrane proteins, avidins, fatty-acid-binding proteins and lysozyme inhibitors and different from crystallins that also consist of two Greek-key motifs. Analysis of the structure and surface properties suggests that Vaps might act as binding proteins involved in interaction with as yet unidentified ligands.

## Supplementary Material

Supporting Information.. DOI: 10.1107/S2053230X14009911/hv5258sup1.pdf

PDB reference: *R. equi* VapB, 4cv7

## Figures and Tables

**Table 1:** Data collection and processing Values in parentheses are for the outer shell.

Data set	Phasing	Refinement
Diffraction source	Microfocus sealed tube	ESRF beamline ID29
Wavelength (Å)	1.5418	0.91985
Temperature (°C)	−173	−173
Detector	Atlas CCD	PILATUS 6M
Crystal-to-detector distance (mm)	52	286
Rotation range per image (°)	0.35 or 0.5	0.3
Total rotation range (°)	1379	360
Space group	*P*6_1_22	*P*6_1_22
*a*, *b*, *c* (Å)	83.55, 83.55, 49.28	83.82, 83.82, 49.13
Mosaicity (°)	0.15	0.10
Resolution range (Å)	15–1.9 (1.95–1.90)	50–1.4 (1.44–1.40)
Total No. reflections	1113900 (26028)	774043 (43740)
No. unique reflections	15146 (1111)†	20584 (1499)‡
Completeness (%)	99.7 (100)	100 (100)
Multiplicity	73.5 (23)†	37 (29)‡
〈*I*/σ(*I*)〉	49.7 (5.6)	26.9 (2.7)
*R*_meas_ § (%)	13.8 (71.6)	9.6 (126.4)
CC_1/2_	100.0 (92.7)	100.0 (85.2)
Overall *B* factor from Wilson plot (Å^2^)	20	22

† Friedel mates separate.

‡ Friedel mates merged.

§ *R*_meas_ as defined in Diederichs & Karplus (1997 ▶): *R*_meas_ = 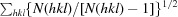 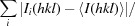 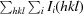.

**Table 2:** Structure solution and refinement Values in parentheses are for the outer shell.

Resolution range (Å)	50–1.4 (1.436–1.400)
Completeness (%)	99.95 (99.87)
No. of reflections	
Working set	18526 (1345)
Test set	2030 (147)
Final *R*_cryst_ (%)	17.0 (22.7)
Final *R*_free_ (%)	19.9 (26.9)
No. of non-H atoms	
Protein	915
Ion	2
Water	107
Total	1024
R.m.s. deviations	
Bonds (Å)	0.024
Angles (°)	2.234
Average *B* factors (Å^2^)	
Protein	20.4
Ion	22.5
Water	29.2
Ramachandran plot	
Most favoured (%)	96.8
Allowed (%)	2.4
Outliers (%)	0.8

## Footnotes

1 Supporting information has been deposited in the IUCr electronic archive (Reference: HV5258).
