# Supplementary material for: Structure of Rhodococcus equi virulence-associated protein B (VapB) reveals an eight-stranded antiparallel β-barrel consisting of two Greek-key motifs
Source: Acta Crystallogr F Struct Biol Commun. 2014 Jun 18;70(Pt 7):866–71. doi: 10.1107/S2053230X14009911 (PMC4089522; doi:10.1107/S2053230X14009911)
Supplement: Supplementary file 1 [file f-70-00866-sup1.pdf]

Acta Crystallographica Section F

Volume 70 (2014)

Supporting information for article:

Structure of *Rhodococcus equi* virulence-associated protein B (VapB) reveals an eight-stranded antiparallel β-barrel consisting of two Greek-key motifs

Christina Geerds, Jens Wohlmann, Albert Haas and Hartmut H. Niemann

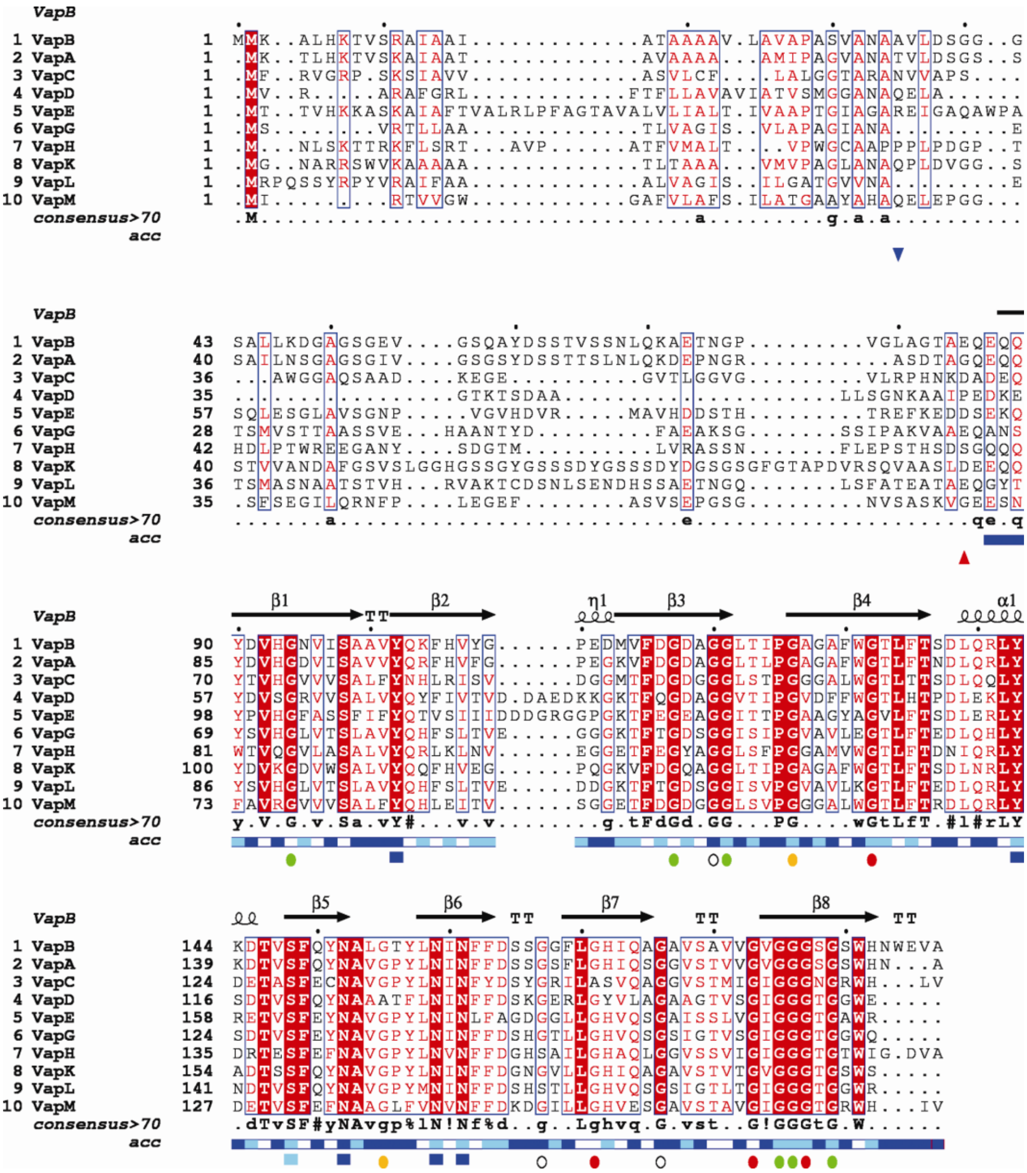

Figure S1 Sequence alignment of 10 Vaps. The blue triangle in the bottom line indicates the first residues of mature VapB after the (predicted) cleavage site of the signal peptidase. The red triangle in the bottom line indicates the N-terminus of the proteinase K resistant VapB fragment. Secondary structure of VapB is indicated above the sequence. The consensus sequence was generated using criteria from MultAlin (uppercase is identity, lowercase is consensus level > 0.5, ! is anyone of IV, , % is anyone of FY, # is anyone of NDQEBZ). Solvent accessibility is displayed below the consensus sequence (blue = accessible; cyan = intermediate; white = buried). Blue squares in the bottom line mark conserved exposed residues whose side chains form hydrogen bonds that presumably stabilize the fold. Ser148 is marked by a cyan square instead, because two hydrogen bonds to neighbouring residues are not direct but water-mediated. Green circles mark conserved glycine residues that fall into the region of the Ramachandran plot allowed or additionally allowed for non-glycine residues and that are presumably conserved due to clashes of a C^{β} atom with neighbouring residues. Orange circles mark conserved glycine residues that fall into the region of the Ramachandran plot that is

forbidden for non-glycine residues. Red circles mark conserved glycine residues that both fall into the region of the Ramachandran plot that is forbidden for non-glycine residues and where addition of a C^{β} atom would cause clashes. Open circles mark conserved glycine residues that do not appear to be conserved due to space constraints, i.e. they fall into the region of the Ramachandran plot allowed or additionally allowed for non-glycine residues and addition of Cβ and further atoms would not cause clashes. Sequences were aligned with *MAFFT* (Katoh & Standley, 2013). The figure was prepared with *ESPript 3* (Gouet *et al.*, 2003).

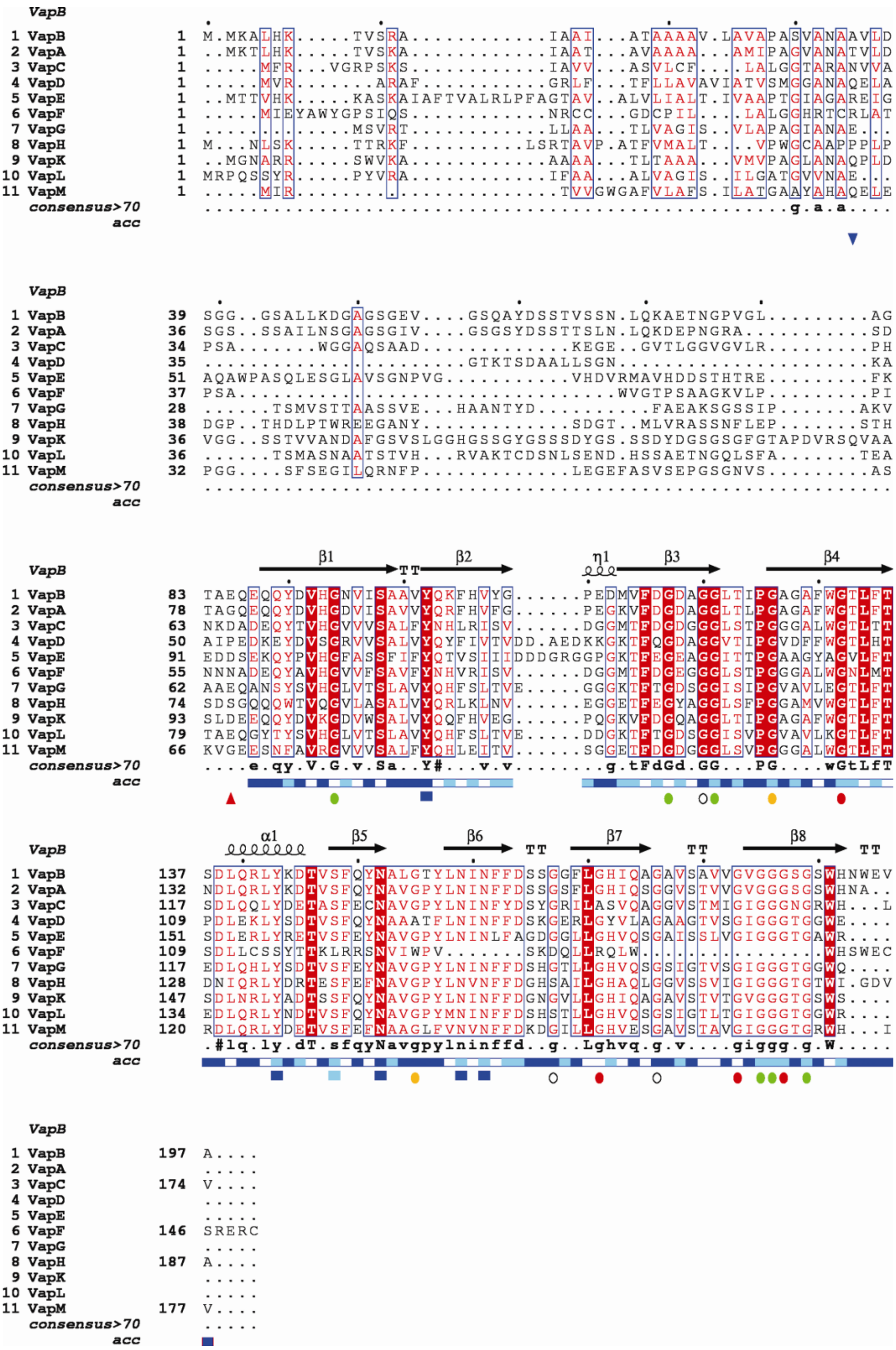

Figure S2 Same as Fig. S1 but additionally including the sequence of VapF. VapF lacks highly conserved sequences of strands β7 and β8 suggesting that it may not form a stable protein of the same fold.
